# Attenuation of radiation toxicity by the phosphine resistance factor dihydrolipoamide dehydrogenase (DLD)

**DOI:** 10.1038/s41598-019-42678-w

**Published:** 2019-04-23

**Authors:** Saad M. Alzahrani, Paul R. Ebert

**Affiliations:** 1 The University of Queensland, School of Biological Sciences, St Lucia, QLD 4072 Australia; 2 King Abdulaziz City for Science and Technology (KACST), Nuclear Science Research Institute (NSRI), P. O. Box 6086, Riyadh, 11442 Saudi Arabia

**Keywords:** Agricultural genetics, Nuclear energy

## Abstract

Phosphine gas is an excellent fumigant for disinfesting stored grain of insect pests, but heavy reliance on phosphine has led to resistance in grain pests that threatens its efficacy. Phosphine-resistance was previously reported to be mediated by the enzyme DLD. Here we explore the relationship between phosphine toxicity and genotoxic treatments with the goal of understanding how phosphine works. Specifically, we utilized mutant lines either sensitive or resistant to phosphine, gamma irradiation or UV exposure. The phosphine-resistance mutation in the enzyme of energy metabolism, dihydrolipoamide dehydrogenase exhibited cross-resistance to UV and ionizing radiation. Two radiation-sensitive mutants that are defective in DNA repair as well as a mutant that is defective in the activation of the DAF-16 stress response transcription factor all exhibit sensitivity to phosphine that exceeds the sensitivity of the wild type control. A radiation resistance mutation in *cep-*1, the p53 orthologue, that is deficient in double strand break repair of DNA and is also deficient in apoptosis causes radiation-resistance results but sensitivity toward phosphine.

## Introduction

The most widely used fumigant globally is hydrogen phosphide (PH_3_), commonly known as phosphine. This gas is an ideal fumigant for the control of insect pest infestations in stored commodities, due to the low cost of application, ease of use and lack of chemical residue, as well as the fact that it does not affect seed viability^1^. Residue and environmental risks associated with sulfuryl fluoride and methyl bromide have left phosphine as the only general use fumigant^2,3^. The heavy reliance on phosphine has led to the selection of strong resistance against phosphine among major insect pests of grain, including the flat grain beetle *Cryptolestes ferrugineus*, the lesser grain borer *Rhyzopertha dominica*, the rust red flour beetle *Tribolium castaneum*, the psocids *Liposcelis bostrychophila*, *L. bostrychophila*^4^, and the rice weevil *Sitophilus oryzae*^4–6^.

Mutations in the dihydrolipoamide dehydrogenase gene (called *dld-1* in *Caenorhabditis elegans* and *rph*2 in pest insects) cause phosphine resistance in insects and in the nematode *C. elegans*. The DLD enzyme participates in the regulation of the rate of energy metabolism and the *dld-1*(*wr4*) resistance mutation in *C. elegans* is associated with a suppressed metabolic rate^7^. This is consistent with the proposed mechanism of toxicity, namely that phosphine initiates oxidative stress in exposed organisms due to the induced production of reactive oxygen species as a byproduct of energy metabolism^1,8^.

The threat of phosphine resistance necessitates the development of alternative methods of pest control, which could include the use of ionizing radiation. Ionizing radiation has gained an excellent reputation in pest management, and has been suggested as an alternative to methyl bromide^9,10^. In addition, gamma irradiation is currently used globally as a quarantine treatment for stored commodities. In the USA, Follett^11^ reported that 120 Gy of gamma radiation is sufficient to disinfest rice from the rice weevil *S. oryzae* adults. Also, adult mortality was immediate after exposure to doses of gamma radiation, of 300 and 500 Gy^12^. Also, the dose 300 Gy has caused complete inhibition of the development process in the immature stages of stored product beetles.

Ultraviolet radiation has also been tested as a tool for stored product pest management and as a hygiene treatment^13–16^. UV radiation can stop development of the khapra beetle, *Trogoderma granarium*. and can decrease the fecundity of *Oryzaephilus surinamensis* and *T. castaneum*; by 21.5% and 53.6% respectively. In the model organism *C. elegans*, UV exposure can also reduce fecundity by decreasing the number and viability of eggs produced^17^. In addition, exposure to either 30 or 40 J m^−2^ of UV radiation early in development resulted in >97% mortality of wild type nematodes^18^.

Cross-resistance between ionizing radiation and a number of fumigants (carbon disulfide, methyl bromide, ethylene dibromide and ethylene dibromide plus carbon tetrachloride in a 3:1 mixture) was found in *T. castaneum*. Radiation was also found to induce resistance in insects toward subsequent fumigation. However, exposure to the fumigants did not change sensitivity to ionizing radiation when it was administered after fumigation^19^. Phosphine was unique as gamma irradiation did not affect subsequent sensitivity to phosphine fumigation^20^, despite the fact that phosphine resistant individuals of *R. dominica* are more tolerant of ionizing radiation compared to their susceptible counterparts^21^.

In this work, we use the free-living nematode *C. elegans* as a model organism to investigate the toxic effect of ultraviolet and gamma irradiation. We also describe the cross-resistance and cross-hypersensitivity between mutant strains, to the two types of irradiation and phosphine fumigation.

## Results

### Strains

We have used mutant strains of *C. elegans* that exhibit either resistance or sensitivity toward phosphine, UV irradiation or gamma irradiation to explore the relationship between genotoxic stresses and the toxic stress associated with exposure to phosphine. These mutant strains include the phosphine resistant mutant, *dld-1*(*wr4*)^22^, the UV hypersensitive strains SP483 and SP488^17^ and the ionizing radiation hypersensitive strains DW102 and DW103. SP483 is a DNA nuclease involved in resolving chromosomal crossover events prior to cell division, whereas SP488 is defective in the activation of a general stress response mediated through the DAF-16 transcription factor^23^. The DW102 and DW103 strains are defective in each of two subunits of a ubiquitin ligase that direct proteome remodeling essential for double strand DNA break repair^24^. The final strain is CE1255, which is resistant to apoptosis induced by DNA damage.

### Phosphine toxicity

**Figure 1:**
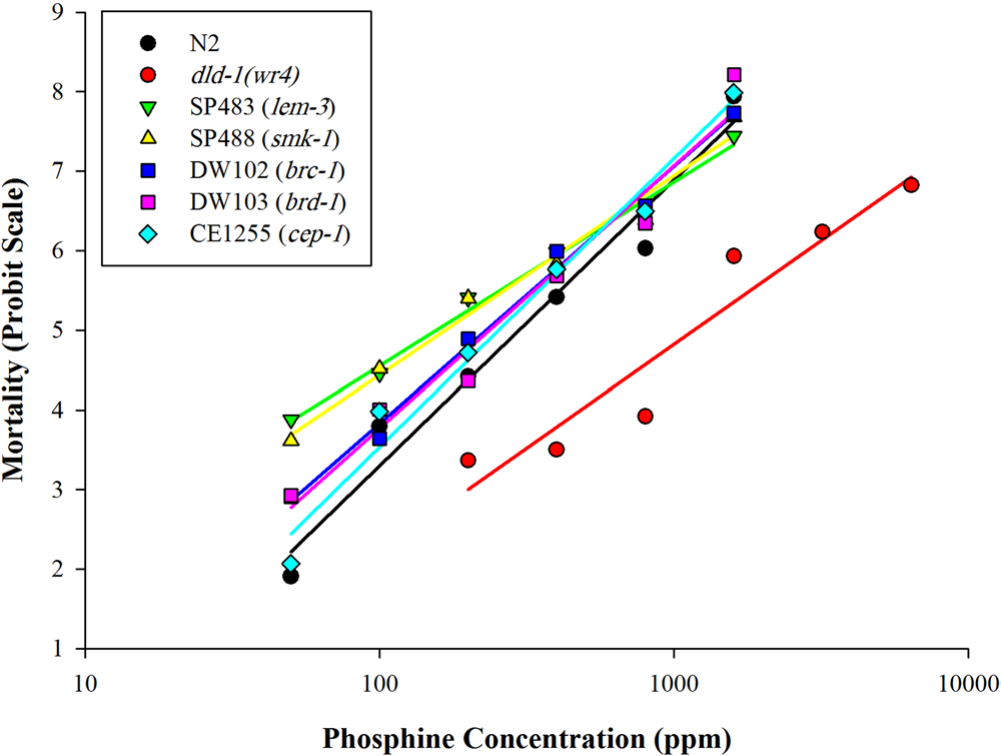
Phosphine-induced mortality in *C. elegans* strains: N2 (wild type)*, dld-1(wr4)* (phosphine-resistant), SP483 & SP488 (UV-sensitive), DW102 & DW103 (ionizing radiation-sensitive), CE1255 (radiation-resistant). Mortality scoring was calculated after 48 hours recovery from 24 hours of phosphine fumigation. Fumigation was repeated three times then averaged for each concentration.

Both phosphine resistant, *dld-1(wr4)*, and susceptible, N2, strains of *C. elegans* exhibit concentration-dependent mortality after exposure to phosphine. The LC_50_ of the phosphine resistant mutant was 4-fold higher than that of the susceptible N2 strain, 1282 ppm, and 302 ppm respectively. The UV hypersensitive strains SP483 and SP488 showed significantly increased sensitivity to phosphine compared to the wild type strain (*P* = *0.0*2*2*) with LC_50_ values of 164 ppm and 174 ppm. The same effect was observed with the ionizing radiation sensitive strains, DW102 and DW103. Their LC_50_ values for phosphine exposure were 225 ppm and 260 ppm, although only DW102 was statistically more sensitive to phosphine than N2 (*P* = *0.043*). The radiation resistant mutant CE1255 did not exhibit cross-resistance to phosphine. In fact, the strain displayed increased sensitivity to phosphine, with an LC_50_ of 239 ppm, which was significantly (*P* = *0.048*) lower than N2 (Fig 1, Table 1).

**Table 1:** LC_50_/LD_50_ values of *C. elegans* strains after either 24 hours phosphine fumigation, UV and gamma irradiation.

Treatment	Strain^†^	LD_50_/LC_50_^‡^	R	Slope ± SE	*X* ^*2*^	*df*
PH_3_ (ppm)	N2	301.88 (270.44–337.06)	0.98	2.89 ± 0.19	6.03	6
	*dld-1(wr4)*	1282.19 (739.32–2142.90)^****^	0.97	3.21 ± 0.23	19.36	5
	SP483	164.02 (92.54–265.55)^*^	0.99	1.99 ± 0.16	19.86	6
	SP488	173.53 (153.05–195.56)^*^	0.99	2.39 ± 0.15	6.47	6
	DW102	225.20 (203.24–249.44)^*^	0.99	3.32 ± 0.23	4.56	6
	DW103	260.41 (233.42–290.44)	0.98	2.93 ± 0.19	9.32	6
	CE1255	238.67 (215.21–264.15)^*^	0.99	3.06 ± 0.18	2.81	6
UV (J cm^−2^)	N2	18.35 (13.40–22.34)	0.97	2.47 ± 0.20	12.95	7
	*dld-1(wr4)*	31.07 (25.48–36.54)^****^	0.97	2.84 ± 0.19	16.81	8
	SP483	15.06 (13.54–16.74)	0.98	1.96 ± 0.11	13.86	10
	SP488	13.84 (12.67–15.13)	0.99	2.68 ± 0.15	11.97	9
	CE1255	41.31 (35.43–47.79)^****^	0.97	4.18 ± 0.33	16.68	7
γ (Gy)	N2	401.23 (362.54–443.24)	0.99	3.29 ± 0.21	3.61	7
	*dld-1(wr4)*	654.58 (603.50–709.45)^****^	0.99	5.20 ± 0.44	0.72	4
	DW102	333.67 (301.52–368.74)	0.99	3.33 ± 0.21	1.62	7
	DW103	344.28 (308.78–383.16)	0.99	2.87 ± 0.17	5.77	7
	CE1255	601.67 (551.33–654.74)^****^	0.99	4.44 ± 0.32	7.33	5

^†^N2 (wild type), *dld-1(wr4)* (phosphine-resistant), SP483 & SP488 (UV-sensitive), DW102 & DW103 (ionizing radiation-sensitive) and CE1255 (resistant to radiation-induced apoptosis).

^‡*^p < 0.05, ^****^p < 0.0001

Values were computed from probit analysis for each strain and treatment. One way ANOVA followed by Dunnett**’**s multiple comparison test was performed to identify significant differences in LC_50_ values due to exposure of each treatment between the wild type and the other strains.

### UV Radiotoxicity

**Figure 2:**
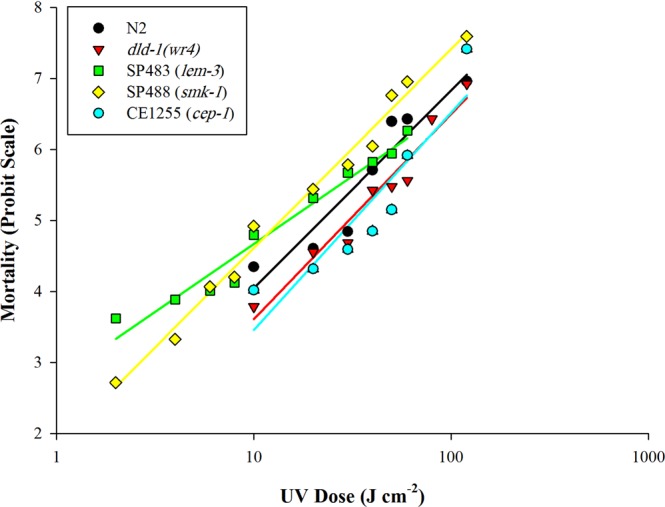
UV-induced mortality in *C. elegans* strains: N2 (wild type)*, dld-1(wr4)* (phosphine-resistant), SP483 & SP488 (UV-sensitive), CE1255 (radiation-resistant). Mortality scoring was scored after 48 hours recovery from UV exposure. UV treatment was repeated three times then averaged for each dosage.

We also monitored both mortality and growth responses of *C. elegans* to ultraviolet radiation. Mortality monitored at 48 and again at 72 hours after UV exposure was dose-dependent. Using log dose probit (LDP) analysis, the median lethal dose (LD_50_) at 48 hours was 18 J cm^−2^ for the wild type strain and 31 J cm^−2^ for the *dld-1(wr4)* mutant (Fig. 2, Table 1). Thus, the *dld-1(wr4)* mutant that was initially selected for its phosphine resistance phenotype, also exhibits 1.7-fold cross-resistance to UV radiation (*P* = *0.0002*).

The UV-sensitive strains SP483 and SP488 showed an apparent sensitivity to UV radiation when mortality was assayed, with LD_50_ values for UV-radiation exposures of 15 J cm^−2^ and 14 J cm^−2^, for the two mutants, respectively, versus 18 J cm^−2^ for the wild type strain. These values did not reach the level of statistical significance relative to the wild type strain (*P* > *0.05*). CE1255 showed a significant increase in resistance to UV-radiation with an LD_50_ of 41 J cm^−2^, a 2.3-fold increase relative to wild type (*P* < *0.0001*) (Fig. 2, Table 1).

UV radiation also causes a dose-dependent inhibition of the growth rate, which we quantified for each of the strains. Under normal conditions, the average length of the five strains was similar, with the exception of SP483, which is significantly shorter than the wild type strain at (*P* = 0.05). The average length after a 48 hours recovery from exposures ranging from 10 to 60 J cm^−2^, did not differ significantly between the wild type strain and the *dld-1(wr4)* mutant except at 60 J cm^−2^ (Table S1). The sizes of the other mutants differed significantly from the wild type strain at most doses. At the highest dose of 60 J cm^−2^, the DNA damage response deficit of strain SP483 resulted in a 70% reduction in the average body length from 0.64 ± 0.01 mm to 0.19 ± 0.03 mm, which is consistent with the reported UV sensitivity phenotype of this strain^25,26^. The SP488 strain exhibited a 79% reduction in the average animal length from 0.91 ± 0.07 to 0.19 ± 0.05 mm at a UV exposure of 60 J cm^−2^ (*P* < *0.0001*). This reflects the disrupted activation of the DAF-16 stress response transcription factor due to the *smk-1* mutation in this strain.

The UV-induced reduction in the average length of wild type animals at 48 hours after exposure to 60 J cm^−2^ was 0.38 ± 0.16 mm. This represents a 59% reduction from the length of control animals that had not been exposed to UV radiation, 0.92 ± 0.07 mm. In contrast, the CE1255 strain with the *cep-1* mutation was statistically similar to the wild type animals after exposure to the same dose with an average length of 0.22 ± 0.17 mm. This represents a 77% reduction from the length of similarly treated wild type animals, 0.95 ± 0.01 mm (Table S1). On the other hand, the *dld-1(wr4)* mutants showed a significant increase in tolerance to the UV-inhibition of growth compared to the wild type. The average length decreased from 0.92 ± 0.07 mm to 0.56 ± 0.13 mm after 60 J cm^−2^ of UV radiation, which is only a 39% decrease. Relative to unexposed nematodes in each strain, the dose at which 50% growth inhibition would occur after 48 hours was estimated to be 52, 70, 37, 11 and 35 J cm^−2^ for strains N2, *dld-1(wr4)*, SP483, SP488 and CE1255 respectively (Table 2). As anticipated, the magnitude of the dose-dependent reduction in growth was greater after 72 hours but the data were consistent with the 48 hour data (Table 2).

**Table 2:** ID_50_ values of *C. elegans* strains after 48 and 72 hours from exposure to UV and gamma irradiation.

Time post exposure	Treatment	Strain^†^	ID_50_^‡^	R	Slope ± SE	*X* ^*2*^	*df*
48 hrs	UV (J cm^−2^)	N2	51.63 (47.56–57.40)	0.99	3.71 ± 0.42	0.18	5
		*dld-1(wr4)*	69.85 (60.64–86.49)^***^	0.99	2.90 ± 0.37	1.72	6
		SP483	36.60 (32.62–41.52)^**^	0.98	2.12 ± 0.23	4.15	6
		SP488	10.50 (6.51–13.92)^****^	0.96	1.35 ± 0.22	2.92	5
		CE1255	35.11 (31.08–39.21)^**^	0.97	2.38 ± 0.35	2.95	5
	γ (Gy)	N2	276.00 (221.15–355.12)	0.96	1.20 ± 0.14	3.61	5
		*dld-1(wr4)*	320.84 (243.26–455.75)	0.99	0.96 ± 0.14	0.05	5
		DW102	297.17 (218.61–438.03)	0.97	0.85 ± 0.14	2.72	5
		DW103	246.68 (128.77–570.69)	0.95	1.60 ± 0.15	12.78	5
		CE1255	489.37 (331.01–1567.23)	0.97	1.67 ± 0.17	11.48	5
72 hrs	UV (J cm^−2^)	N2	61.20 (54.39–72.22)	0.99	2.98 ± 0.35	2.20	6
		*dld-1(wr4)*	66.15 (57.40–83.39)	0.96	2.77 ± 0.41	5.82	5
		SP483	48.06^§^	0.87	1.86 ± 0.23	23.54	
		SP488	10.83 (5.49–15.21)^****^	0.93	1.03 ± 0.20	3.68	6
		CE1255	64.52 (54.96–81.27)	0.96	2.21 ± 0.28	7.67	5
	γ (Gy)	N2	223.28 (171.94–294.31)	0.99	1.02 ± 0.14	0.42	5
		*dld-1(wr4)*	290.63 (229.88–382.56)	0.99	1.13 ± 0.14	1.98	5
		DW102	205.43 (158.38–267.37)	0.99	1.04 ± 0.14	1.52	5
		DW103	421.54 (355.28–516.91)^***^	0.96	1.80 ± 0.17	6.17	5
		CE1255	444.46 (369.12–558.06)^***^	0.99	1.67 ± 0.17	3.09	5

^†^N2 (wild type), *dld-1(wr4)* (phosphine-resistant), SP483 & SP488 (UV-sensitive), DW102 & DW103 (ionizing radiation-sensitive) and CE1255 (resistant to radiation-induced apoptosis).

^‡*^p < 0.05, ^**^p < 0.005^, ***^p < 0.001 and ^****^p < 0.0001.

^§^Confidence limits could not be computed because G-test is >0.4.

Values were computed from probit analysis for each strain and treatment. The growth-inhibitory effect of each treatment is compared to the unexposed animals as growth reduction percentages. A comparison with one way ANOVA followed by Dunnett**’**s multiple test was performed to identify significant differences in ID_50_ values due to exposure of radiation treatments between the wild type and the other strains.

### Gamma Radiotoxicity

**Figure 3:**
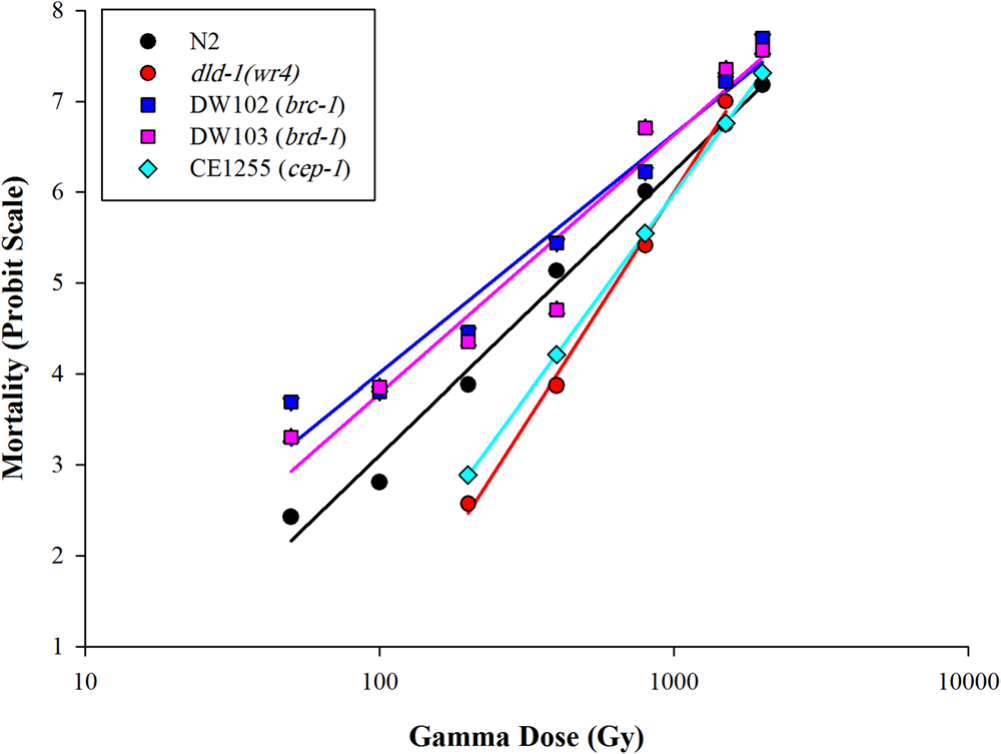
Gamma-induced mortality for the N2 (wild type)*, dld-1(wr4)* (phosphine-resistant), DW102 & DW103 (ionizing radiation-sensitive), CE1255 (radiation-resistant). Mortality scoring was after 48 hours recovery from exposing L_1_ nematode to doses of gamma radiation. Irradiation was repeated three times then averaged for each dose (Gy).

Forty-eight hours after exposure of L_1_ nematodes to gamma radiation, dose-dependent mortality was apparent (Fig. 3, Table 1). The *dld-1*(*wr4*) mutant exhibited tolerance to gamma radiation, with an LD_50_ of 655 Gy, compared to 401 Gy for the wild type N2 strain (*P* < *0.0001*). In contrast, the LD_50_ values for the radiation sensitive mutants were 334 Gy for DW102 (P < 0.044) and 344 Gy for DW103 (P > 0.05). The gamma radiation resistant strain, CE1255 was significantly more tolerant than the wild type strain with an LD_50_ of 602 Gy compared to 401 Gy (*P* < *0.0001*). The level of tolerance of CE1255 was statistically indistinguishable from that of the *dld-1*(*wr4*) mutant.

As with UV, ionizing radiation inhibited the growth of the nematodes in a dose-dependent manner, as determined after a 48 hour recovery period following the exposure (Fig. 3, Table 2). In the absence of exposure to gamma radiation, the growth of the mutant strains is statistically indistinguishable from growth of the wild type strain. The *dld-1(wr4)* mutant and the DW102 and DW103 strains each responded to the growth-inhibition induced by gamma-radiation in a similar manner with no significant difference between the mutants and the wild type nematodes across all doses (Table S2). Also, the ID_50_ values of these strains 297, 247 and 276 Gy were statistically indistinguishable from that of the wild type strain (Table 2). On the other hand, the radiation-resistant strain CE1255 was more tolerant of gamma radiation, resulting in less growth inhibition than observed for the wild type strain throughout dose range of the experiment, with the exception of the most extreme exposure of 800 Gy. The dose required to achieve 50% growth inhibition of CE1255 was twice that of the wild type nematodes (Table 2). Similar results were observed after 72 hours post exposure (Tables 2 and S2).

## Discussion

While phosphine fumigation is the most common means of disinfesting grain, irradiation treatment for disinfestation has been employed in countries that include Saudi Arabia, Brazil, China, India, Russia, France, Turkey and the United States^27^. The co-existence of phosphine fumigation and ionizing radiation as pest management tools in the grain storage system raises the need to understand how the two treatments act and how they potentially interact.

Phosphine is a reducing agent that interferes with cellular respiration. Exposure to phosphine can initiate oxidative stress by excessive production of reactive oxygen species (ROS)^1,8^. ROS are generated naturally as a byproduct of metabolic electron transfer reactions, notably from the mitochondrial electron transport chain (ETC). ROS react aggressively with other molecules including proteins, lipids and DNA eventually leading to cell death^28–30^.

Phosphine-resistance in *C. elegans* and insects is mediated by genetic modification of DLD^31^. The dihydrolipoamide dehydrogenase enzyme is a subunit of four enzyme complexes that feed metabolites of carbohydrate and amino acids into aerobic energy metabolism^32^. In *C. elegans*, a mutation in the *dld-1* gene causes not only phosphine resistance but also a 75% decrease in aerobic respiration, monitored as a decrease in oxygen consumption^7,33^. Aerobic respiration is essential to phosphine toxicity^33–37^ and is a significant source of ROS. It is likely that the resistance of the *C. elegans* mutant is mediated by a decrease in ROS generation on exposure to phosphine as a direct result of the suppressed metabolism (Table 1).

The primary injurious effect of UV and ionizing irradiation on living organisms is DNA-damage. This includes single or double-strand DNA breaks (SSBs, DSBs)^38,39^. *lem-3* is a DNA nuclease that aids in the resolution of inter-chromosomal crossover events prior to cell division as well as protecting against the genotoxic effect of UV radiation, X-rays and other DNA-damaging chemicals^17,40^. The other UV sensitive mutation that was used in this report was in the smk-1 gene, which facilitates gene regulation by DAF-16. DAF-16 is a general stress response transcription factor that promotes survival following a range of stresses including UV exposure. Interestingly, both mutants exhibit cross sensitivity to the fumigant phosphine.

The *brc-1* and *brd-1* genes encode proteins that form a heterodimeric ubiquitin ligase that participates in proteome remodelling toward DSB repair^24,41,42^. Mutations in these genes cause increased sensitivity to ionizing radiation-induced DNA damage as well as increased sensitivity to phosphine exposure. The increased sensitivity to phosphine gas displayed by each of the four mutants (Table 1) clearly demonstrates that the radiation defense responses also protect against phosphine. The SP483 strain that carries the mutation in the *lem-3* gene is the only mutation that disrupts a specific DNA repair function, which suggests that phosphine is also a DNA-damaging agent. This is consistent with the report of phosphine causing oxidation of DNA in the brain tissue of rats that had been exposed to phosphine orally^29^. The other three mutations influence a broad range of stress response genes, so do not specifically implicate DNA damage as the toxic product of phosphine exposure.

The final mutant strain used in this study, CE1255, carries a mutation in the *cep-1* gene, which is the *C. elegans* orthologue of the mammalian p53 gene. The p53 protein guards against potentially oncogenic mutations by halting progression of the cell cycle following DNA replication to allow any DNA replication errors or DNA damage to be repaired prior to cell division. In addition to its role as the cell cycle checkpoint protein that promotes radioresistance by facilitating DNA repair^43^, p53 can also protect an organism from cancer by promoting the elimination of cells through apoptosis if they suffer DNA damage is too severe to be repaired. The *cep-1* allele in CE1255 causes resistance to radiation-induced apoptosis. While this mutation promotes survival of *C. elegans* following exposure to radiation, it did not result in cross-resistance to phosphine. This contradicts the notion that the toxicity of phosphine results in part from stimulating apoptosis via mitochondrial insult^44,45^. However, the studies that suggested a role for apoptosis in phosphine toxicity were performed on rats with extremely high doses of aluminium phosphide rather than the much lower concentrations of phosphine gas that we employed. Our study also avoided the confounding effects of aluminiumat were administered in the experiments with rats^44,45^. As a result, our experimental conditions seem to have been insufficient to trigger apoptotic cell death.

Despite the lack of cross-resistance of the p53 mutant to phosphine as well as radiation, the *dld-1* mutant that is resistant to phosphine was also cross-resistant to both UV and ionizing radiation. Phosphine toxicity is mediated through the generation of reactive oxygen species, whereas the *dld-1* mutation alleviates phosphine-induced oxidative damage in exposed animals^7^. Similarly, radiation causes oxidative stress by triggering generation of ROS in damaged cells^46–48^. The ability of the phosphine resistant mutant, *dld-1*, to provide cross-protection against radiation-induced damage suggests that oxidative damage also contributes to radiation-induced mortality in *C. elegans*^1,8^. The cross-resistance that we observe in *C. elegans* was previously observed in the lesser grain borer *R. dominica*, which is an insect pest of stored grain. In these experiments, a phosphine resistant strain of *R. dominica* was more resistant to ionizing radiation than their phosphine-sensitive counterparts. While the resistance factor in these insects was not identified, the authors suggest that the phosphine resistant insects likely have the genetic ability to counter oxidative damage caused by phosphine, and can tolerate exposure to ionizing radiation since it has been reported to cause oxidative stress^21^. In support of their conclusions, antioxidants have been found to protect against phosphine exposure^28,29^. The simplest conclusion is that the cross-resistance of the *dld-1* mutation results from protection against oxidative damage, which is a mechanism of action shared between phosphine gas, gamma radiation and UV radiation.

UV radiation induces the generation of reactive radicals that cause oxidative damage to the macromolecules in the cell including DNA. One of the genes that plays a major role in the oxidative stress response in C. elegans is *daf-2*, which encodes a negative regulator of the stress response transcription factor DAF-16. Mutation of the *daf-2* gene results in an increase in expression of stress response genes mediated by the DAF-16 transcription factor, as well as an increase in the tolerance to UV-exposure. Conversely, the SP488 strain used in this study is defective in a positive regulator of DAF-16 mediated gene expression, resulting in attenuation of the DAF-16 stress response^18,49^. The result is sensitivity to UV-exposure and to phosphine exposure as well.

We also monitored growth inhibition in response to radiation exposure. This inhibition is most likely due to cell cycle arrest as a result of DNA damage as the cell cycle arrest mutant CE1255 had a normal growth rate. The cell cycle arrest is a primary defense mechanism in living organisms against radiation damage^38^ as stopping the cell cycle allows the cell to repair the DNA, preventing the replication and inheritance of damaged DNA by the daughter cells. In our results, this stoppage was expressed in the exposed nematodes as a growth inhibition where the surviving nematodes were shorter than the unexposed worms due to a delay in their growth. Thus, a delay in growth can actually be a benefit for survival. This association between delayed growth and enhanced survival was seem for some, but not all of the mutants used in this study.

## Conclusion

Radiotoxicity and phosphine toxicity both involve oxidative stress. Phosphine resistant animals are able to resist radiation-induced damage. However, a mutation that results in resistance to radiation-induced apoptosis and cell cycle inhibition does not provide resistance to phosphine. Likewise, mutations that are defective in repair of double and single stranded breaks to DNA are sensitive to gamma radiation, whereas phosphine susceptibility is not affected. In contrast, mutation of an activator of the general stress response transcription factor, DAF-16 causes greater susceptibility to both UV light and phosphine.

## Materials and Methods

### Nematode strains and culture conditions

We used six mutant strains of *C. elegans* in these studies: *dld-1*(*wr4*), which is resistant to the fumigant phosphine^22^. SP483 and SP488 are both hypersensitive to UV radiation^17^. DW102 and DW103 are both hypersensitive to gamma radiation. CE1255 is resistant to both UV and gamma radiation. SP483, DW102 and DW103 are deficient in repair of radiation induced damage to DNA whereas CE1255 is resistant to apoptosis induced by DNA damage. In contrast, SP488 is defective in the activation of a general stress response transcription factor, that is not specifically linked to DNA damage. The mutant strains are available through the *C. elegans* Genetic Center (CGC), which is funded by NIH Office of Research Infrastructure Programs (P40 OD010440).

The nematodes were maintained on NGM agar plates at 20 °C. All experiments were carried out at 20 °C as well. The protocol for maintaining *C. elegans* was followed as described in wormbook^50^. Age-synchronization was obtained by harvesting eggs from gravid adults using alkaline sodium hypochlorite. Eggs were maintained with gentle agitation in M9 buffer for 18–20 hours to allow them to hatch. They enter L_1_ diapause in the absence of food and begin synchronized growth when transferred to fresh NGM agar plates (0.3% NaCl, 0.25% peptone, 5 mg/ml cholesterol, 1 mM CaCl_2_, 1 mM MgSO_4_, 1.7% agar) seeded with a lawn of OP50 bacteria (*Escherichia coli*) as a food source.

### UV exposure

According to Hartman^17^ and our preliminary trials, it is relatively difficult to obtain results from irradiating later stages of *C. elegans* due to the time required for the phenotype to develop and the complication of progeny being produced during that period. Therefore, L_1_ stage nematodes on NGM agar plates were treated with a dose range of UV as follows 0, 2, 4, 6, 8, 10, 20, 30, 40, 50 60 and 120 J cm^−2^ using (XLE-Series UV crosslinker, Spectronics Co.) as described in Hartman, 1984^18^. After each treatment, the nematodes were transferred to a 20 °C incubator to recover for 48 hours prior to mortality assessment. Each experiment was repeated three times, and each trial contained two technical replicates per strain for each treatment.

### Ionizing radiation

Synchronized L_1_ worms on NGM agar plates were irradiated according to Johnson and Hartman, 1988^51^, with dose range of gamma rays 0, 50, 100, 200, 400, 800, 1500, 2000 Gy. A cobalt-60 Gammacell-220 irradiator (Atomic Energy of Canada Ltd.) in the School of Chemistry and Molecular Biosciences at The University of Queensland was used as the gamma rays source. After each treatment, the nematodes were transferred to a 20 °C incubator to recover for 48 hours prior to mortality assessment. Each experiment was repeated three times, and each trial contained two technical replicates per strain for each treatment.

### Phosphine fumigation

The phosphine fumigation was carried out at 20 °C, according to the fumigation protocol described in Valmas and Ebert, 2006^52^. The plates were placed in air-tight desiccators into which a measured amount of phosphine gas was injected. In all cases, the volume of gas that was injected into the chamber was less than 0.2% of the volume of the chamber. Phosphine fumigations were carried out at 0, 50, 100, 200, 400, 800, 1600, 3200 and 6400 ppm for 24 hours. Following the fumigation, the nematodes were transferred to fresh air to recover for 48 hours prior to length measurement and mortality assessment via WormScan^53^. Each experiment was repeated three times, and each trial contained two technical replicates per strain for each treatment.

### Data acquisition via WormScan

Each experiment was repeated three times, and each trial contained two technical replicates per strain for each treatment. The WormScan system was utilized for phenotype assessment as described in^53,54^. Briefly, the treated worms in six centimeters petri dishes were scanned using transmission photo scanners. Individuals that did not move in response to a light stimulus across a ten minutes period were scored as dead.

The WormScan system was also used to assess growth inhibition by measuring the length of all surviving animals at both 48 and 72 hours after irradiation relative to the growth of non-irradiated controls (Table 2) or the irradiated wild type strain (Tables S1 and S2)^53^.

### Statistical analysis

Probit analysis^55^ was carried out using (LdP Line, copyright 2000 by Ehab Mostafa Bakr, Cairo, Egypt) to calculate the median lethal concentration/dose (LC_50_/LD_50_) and the 95% confidence intervals. One way ANOVA followed by Dunnett’s multiple comparisons was carried out to determine the statistical significance in the response of strains to each treatment. Probit data were plotted using SigmaPlot version 10.0, from Systat Software, Inc., San Jose California USA^56^.

Two-way ANOVA followed by Dunnett’s multiple comparison test was carried out to determine the significance of the differences using GraphPad Prism (Prism version 7.00 for Windows, GraphPad Software, La Jolla California USA, www.graphpad.com). Inhibition was calculated as the dose required to inhibit growth by 50% (ID_50_) at either 48 or 72 hours post exposure.

## Supplementary information

Supplementary tables

## Data Availability

Any data can be requested from Paul Ebert^1^ (p.ebert@uq.edu.au).
