# Supplementary material for: Attenuation of radiation toxicity by the phosphine resistance factor dihydrolipoamide dehydrogenase (DLD)
Source: Sci Rep. 2019 Apr 23;9:6455. doi: 10.1038/s41598-019-42678-w (PMC6478721; doi:10.1038/s41598-019-42678-w)
Supplement: Supplementary file 1 — Supplementary tables [file 41598_2019_42678_MOESM1_ESM.pdf]

Attenuation of radiation toxicity by the phosphine resistance factor dihydrolipoamide dehydrogenase (DLD)

Saad Alzahrani[1,2] and Paul R. Ebert[1,*]

[1] The University of Queensland, School of Biological Sciences, St Lucia, QLD, 4072 Australia

[2] King Abdulaziz City for Science and Technology (KACST), Nuclear Science Research Institute (NSRI), P. O. Box 6086, Riyadh 11442, Saudi Arabia

[*] Corresponding author

Table S1: Inhibition of growth-induced by UV after 48hrs and 72hrs from irradiating L_1 stage. Two-way ANOVA followed by Dunnett's multiple comparisons test to compare the average animal length between the wild type strain and the other strains.

UV Dose (J cm^{-2})	Average animal length (mm±SD)†				
	**N2**‡	***dld-1(wr4)***	**SP483**	**SP488**	**CE1255**
	**48 hrs**				
0	0.92±0.07	0.92±0.07	0.64±0.01***	0.91±0.07	0.95±0.01
10	0.86±0.15	0.92±0.18	0.56±0.01***	0.44±0.11****	0.66±0.04*
20	0.86±0.10	0.87±0.08	0.40±0.16****	0.35±0.05****	0.66±0.08*
30	0.74±0.21	0.80±0.18	0.43±0.23***	0.34±0.01****	0.57±0.06
40	0.62±0.17	0.70±0.07	0.23±0.23***	0.32±0.02****	0.43±0.16*
50	0.46±0.13	0.57±0.10	0.33±0.06	0.10±0.14****	0.38±0.21
60	0.38±0.16	0.56±0.13*	0.19±0.03*	0.19±0.05*	0.22±0.17
	**72 hrs**				
0	1.39±0.15	1.35±0.10	0.98±0.03****	1.03±0.04***	1.12±0.03*
10	1.37±0.19	1.26±0.14	0.84±0.01****	0.50±0.01****	1.05±0.01**
20	1.30±0.14	1.25±0.09	0.74±0.00****	0.40±0.01****	1.00±0.01**
30	1.15±0.27	1.18±0.24	0.70±0.13****	0.37±0.04****	1.01±0.02
40	0.91±0.37	0.85±0.30	0.64±0.01*	0.34±0.03****	0.85±0.04
50	0.81±0.29	0.86±0.22	0.54±0.08*	0.25±0.11****	0.70±0.21
60	0.77±0.28	0.80±0.32	0.25±0.03****	0.18±0.06****	0.50±0.02*

† ****$p < 0.001$ and *$p < 0.05$ are significantly different compared to the wild type (N2).

‡N2 (wild type), *dld-1(wr4)* (phosphine-resistant), SP483 & SP488 (UV-sensitive), DW102 & DW103 (ionizing radiation-sensitive) and CE1255 (resistant to radiation-induced apoptosis).

Table S2: Inhibition of growth-induced by gamma irradiation after 48hrs and 72hrs from irradiating L_1 stage. Two-way ANOVA followed by Dunnett's multiple comparisons test to compare the average animal length between the wild type and the other strains.

γ-Dose (Gy)	Average animal length (mm±SD)†				
	**N2‡**	***dld-1(wr4)***	**DW102**	**DW103**	**CE1255**
	**48 hrs**				
0	0.88±0.08	0.91±0.07	0.83±0.01	0.80±0.01	0.95±0.01
50	0.77±0.13	0.71±0.05	0.65±0.07**	0.64±0.04***	0.87±0.07*
100	0.55±0.05	0.62±0.04	0.51±0.06	0.61±0.07	0.95±0.14****
200	0.46±0.04	0.52±0.04	0.44±0.04	0.49±0.05	0.59±0.03***
400	0.39±0.01	0.43±0.01	0.36±0.04	0.44±0.03	0.53±0.05***
800	0.27±0.04	0.32±0.03	0.32±0.01	-§	0.39±0.02**
	**72 hrs**				
0	1.10±0.02	1.18±0.10	1.15±0.04	0.94±0.01*	1.12±0.03
50	0.72±0.38	0.94±0.14**	0.88±0.20*	0.95±0.06**	1.05±0.14****
100	0.80±0.12	0.80±0.09	0.69±0.17	0.79±0.06	1.00±0.08**
200	0.58±0.12	0.70±0.08	0.54±0.06	0.62±0.02	0.76±0.01*
400	0.45±0.03	0.57±0.06	0.46±0.03	0.52±0.04	0.65±0.04**
800	0.30±0.03	0.32±0.03	0.33±0.02	-§	0.35±0.02

† ****$p < 0.001$ and *$p < 0.05$ are significantly different compared to the wild type (N2).

‡ N2 (wild type), *dld-1(wr4)* (phosphine-resistant), SP483 & SP488 (UV-sensitive), DW102 & DW103 (ionizing radiation-sensitive) and CE1255 (resistant to radiation-induced apoptosis).

§ No surviving animals
